# Phase 2/3 study of intravenous thrombolysis and hypothermia for acute treatment of ischemic stroke (ICTuS 2/3)

**DOI:** 10.1186/cc11271

**Published:** 2012-06-07

**Authors:** Thomas Hemmen, Karen Rapp, Rema Raman, Mauricio Concha, Gregor Brössner, Erich Schmutzhard, Gilda Tafreshi, Vivek Misra, Salvador Cruz-Flores, Rainer Kollmar, David Brown, Irfan Altafullah, Patrik Michel, Andrei Alexandrov, Carlos Smith, Julie Jurf, Mary Jane Hess, James Grotta, Patrick D Lyden

**Affiliations:** 1 UCSD Medical Center, San Diego, CA, USA; 2 Sarasota Memorial Hospital, Sarasota, FL, USA; 3 University Hospital Innsbruck, Austria; 4 Scripps Mercy Hospital, San Diego, CA, USA; 5 University of Texas, Houston, TX, USA; 6 St Louis University, St Louis, MO, USA; 7 University of Erlangen, Germany; 8 Hoag Hospital, Newport Beach, CA, USA; 9 North Memorial Hospital, Robbinsdale, MN, USA; 10 University of Lausanne, Switzerland; 11 University of Alabama, Birmingham, AL, USA; 12 St Joseph Hospital, Tampa, FL, USA; 13 Cedars Sinai Medical Center, Los Angeles, CA, USA

## Background

The ICTuS trial showed feasibility of endovascular hypothermia for acute ischemic stroke [1]; ICTuS L confirmed safety and feasibility of endovascular hypothermia during thrombolysis [2]. The ICTuS 2/3 study seeks to determine whether the combination of thrombolysis and hypothermia is superior to thrombolysis alone for the treatment of acute ischemic stroke. A phase 2 study will include 450 patients to assess the safety of various protocol changes, to demonstrate sufficient recruitment, and to allow an interim analysis for futility. If pre-specified milestones are achieved the study will be enlarged to a 1,800-patient phase 3 efficacy study.

## Methods

This is a prospective, randomized, single-blind, multicenter phase 2/3 study. We aim to include ischemic stroke patients treated within 3 hours of symptom onset with IV tPA (according to FDA or EMEA protocol), NIHSS ≥7 and ≤20, age 22 to 80. Patients are randomly assigned to either hypothermia permissively targeted to 33°C or normothermia. Favorable outcome is defined as a 90-day Modified Rankin score (mRS) of 0 or 1. Secondary outcome measures are: 90-day NIHSS, Barthel Index (BI), mortality, shift analysis of the mRS, global odds ratio of mRS, BI, NIHSS, incidence of symptomatic intracranial hemorrhage and 90-day Montreal Cognitive Assessment. An interim analysis for futility is planned near the end of phase 2. In addition to futility, analyses will assess the frequency of target temperature reached within 6 hours from symptom, pneumonia rate, safety profile of iced saline infusion and study-wide average enrollment rate.

## Results

The study team initiated 11 study sites in the USA and two in Europe. Enrolment began in December 2010. Currently, 37 subjects are enrolled. A full DSMB review of experience to date allowed the study to continue enrollment. A safety review after the first 50 patients is expected in late 2012.

## Conclusions

ICTuS 2/3 is the largest trial of endovascular hypothermia for acute stroke currently running. There appear to be no safety or feasibility concerns.
